# Infrared Photobiomodulation Modulates PpIX Production After Topical Methyl Aminolevulinate in Normal Human Skin: An Exploratory Study

**DOI:** 10.1002/jbio.70262

**Published:** 2026-03-27

**Authors:** Letícia Palombo Martinelli, Natasha Ferreira Mezzacappo, Ana Paula Silva, Priscila Menezes, Vanderlei Salvador Bagnato

**Affiliations:** ^1^ São Carlos Institute of Physics University of São Paulo São Carlos Brazil; ^2^ Emipharma Indústria e Comércio de Produtos Fármacos Ltda São Carlos São Paulo Brazil; ^3^ Biomedical Engineering Texas A&M University College Station Texas USA

**Keywords:** clinical trial, methyl Aminolevulinate, Photobiomodulation, photodynamic therapy, Protoporphyrin IX

## Abstract

Photodynamic therapy (PDT) for skin lesions requires optimized photosensitizer accumulation. Since protoporphyrin IX (PpIX) synthesis is linked to mitochondrial activity, photobiomodulation (PBM) may modulate this process. This exploratory study examined the effects of PBM at 780 or 808 nm on MAL‐induced PpIX in normal human skin. Eleven volunteers received paired‐arm treatments (5, 10, or 15 J/cm^2^). Following 30 min of MAL, PBM was applied, with total incubation lasting 210 min. PpIX was assessed via spectroscopy and widefield imaging. Although mean PBM/no‐PBM ratios were nominally above 1, no statistically significant differences were found among fluences or against unity, primarily due to high interindividual variability (CV up to 35%). These findings suggest that while PBM shows a trend toward modulating photosensitizer accumulation, the response is highly heterogeneous. This study provides a rationale for further investigation into personalized PBM‐assisted PDT protocols to manage biological variability and refine clinical outcomes.

## Graphical abstract

We demonstrate that NIR photobiomodulation can influence PpIX accumulation in normal human skin. Although subject to interindividual variability, the observed trends support PBM as a promising, noninvasive adjuvant to refine PDT protocols and manage photosensitizer production through mitochondrial stimulation.

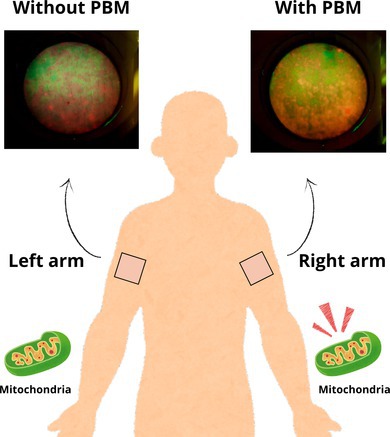

## 1 Introduction

Photobiomodulation (PBM), which uses low‐intensity red and near‐infrared (NIR) light to modulate cellular activity, has been shown to stimulate mitochondrial function, enhance ATP synthesis, regulate reactive oxygen species (ROS) production, and activate key transcription factors [1, 2, 3]. Mitochondria are also fundamentally involved in the biosynthesis of protoporphyrin IX (PpIX) [4, 5]. PpIX is an essential intermediate produced inside mitochondria during heme metabolism, playing a critical role in oxygen transport, energy metabolism, and enzymatic reactions [6]. Additionally, PpIX exhibits strong fluorescence, which allows for its use in noninvasive optical detection and monitoring. Moreover, its ability to generate ROS upon light activation makes it an effective endogenous photosensitizer for photodynamic therapy (PDT) [7, 8]. In healthy mammalian cells, heme biosynthesis is typically well‐controlled, preventing PpIX accumulation [9]. However, the exogenous administration of ALA or its ester derivative, methyl aminolevulinate (MAL), bypasses this regulatory step [9, 10], allowing PpIX to be produced at a rate that surpasses its conversion to heme by ferrochelatase (FECH) enzyme, resulting in a transient accumulation of PpIX [9, 10].

Given the significant role of mitochondria in PpIX generation, we propose that enhancing mitochondrial activity and metabolism through PBM before MAL application promotes higher and more uniform PpIX synthesis in the skin. In vitro studies have shown that PBM can be used to accelerate PpIX production in cancer cells [11, 12]. Applying PBM before PDT may therefore shorten the waiting period between MAL cream application and light exposure, reducing the overall treatment time. This approach has the potential to improve patient comfort while increasing clinical efficiency. Because NIR light penetrates deeper into the skin, PBM has the potential to stimulate PpIX production in layers that are not fully reached by MAL alone. However, before testing this approach in tumors, it is essential to establish whether PBM can indeed enhance PpIX synthesis in normal skin. To our knowledge, no previous studies have investigated this effect in healthy human tissue, only in cell culture [11, 13] or chicken embryo chorioallantoic membrane [11].

Therefore, the present study was designed as a first step, aiming to evaluate the influence of PBM on PpIX production in normal skin across different phototypes. Demonstrating an increase under these conditions provides a strong rationale to expect an even greater enhancement in malignant tissue, where metabolic activity is typically higher [14, 15]. Ultimately, this strategy could benefit future PDT protocols for skin cancers by improving photosensitizer accumulation and treatment efficacy.

## 2 Materials and Methods

This study was approved by the institutional ethics committee and registered in Plataforma Brasil (CAAE: 86365825.8.0000.8148), the official Brazilian system for human research ethics. A total of 11 healthy volunteers (range from 22 to 46 years old) were recruited and randomly allocated into six experimental groups (*n* = 5 per group), according to PBM wavelength (780 or 808 nm) and light fluence employed during the PBM (5, 10, or 15 J/cm^2^), as follows: G1 (780 nm, 5 J/cm^2^), G2 (780 nm, 10 J/cm^2^), G3 (780 nm, 15 J/cm^2^), G4 (808 nm, 5 J/cm^2^), G5 (808 nm, 10 J/cm^2^), and G6 (808 nm, 15 J/cm^2^)—Table 1.

**TABLE 1:** Experimental design showing the photobiomodulation parameters for each group. Each group was defined by wavelength and fluence, with irradiation applied to the four vertices of a 2 × 2 cm marked square on the forearm. Irradiance and power output were verified using a calibrated power meter (Model PM160T‐HP, Thorlabs Inc., Newton, NJ, USA).

Groups	PBM wavelength (nm)	Fluence (J/cm^2^)*	Irradiation time (s)*	Irradiance (mW/cm^2^)	Spot area (cm^2^)	Power output (mW)
G1	780	5	20	250	0.04	10% ± 20%
G2	780	10	40	250	0.04	10% ± 20%
G3	780	15	60	250	0.04	10% ± 20%
G4	808	5	15	333.3	0.03	10% ± 20%
G5	808	10	30	333.3	0.03	10% ± 20%
G6	808	15	45	333.3	0.03	10% ± 20%

* Fluence and irradiation time were delivered at each of the four points marked within the square area.

Some volunteers participated in more than one experimental group; however, a minimum interval of 1 week was maintained between sessions to ensure that no carryover effects or interference influenced the analyses and outcomes [16]. For each participant, a 2 × 2 cm square was demarked on the inner forearm. The left arm served as the control (no PBM exposure), while the right arm received PBM applied at vertices of the marked square.

**FIGURE 1:**
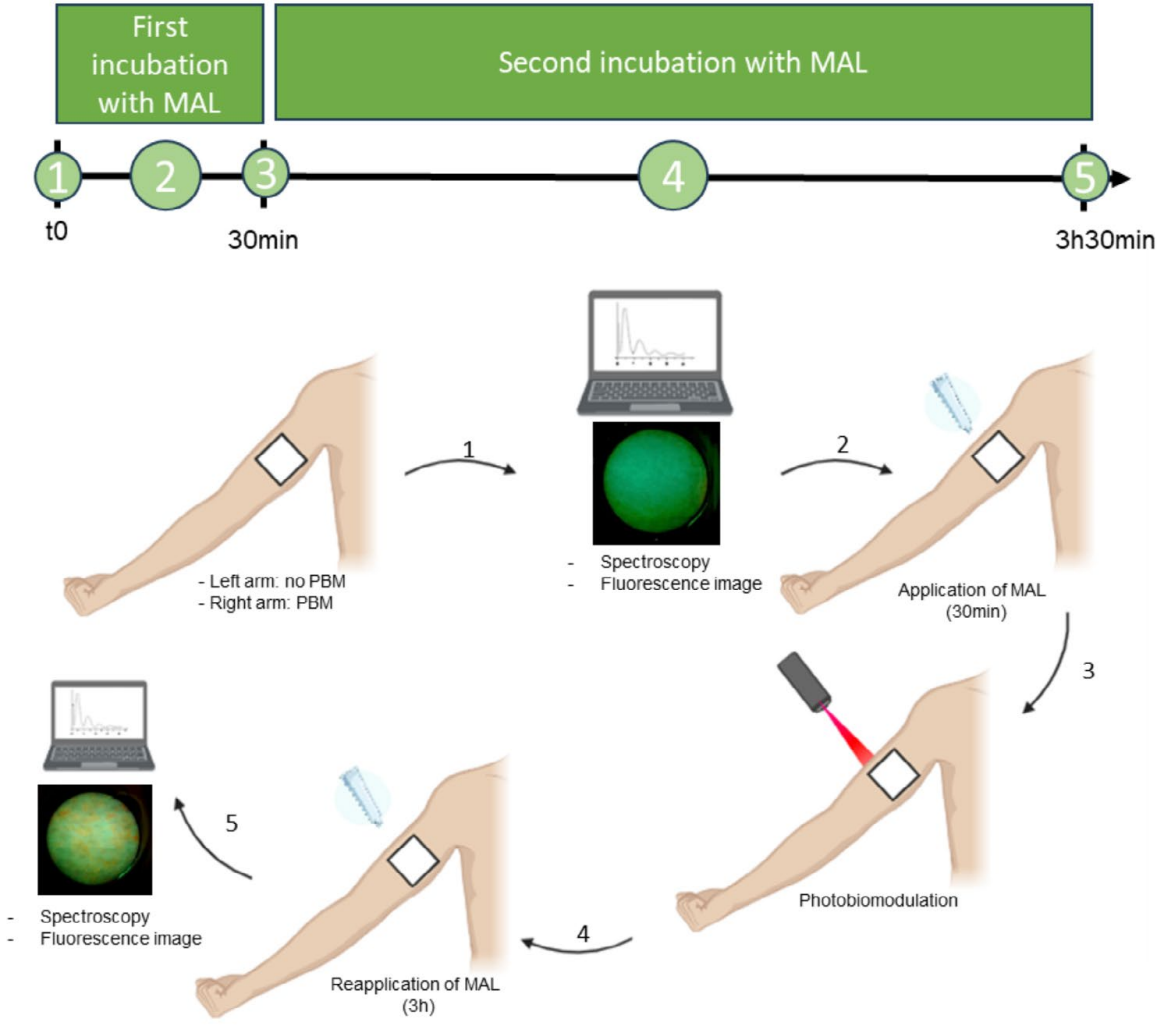
Timeline and schematic representation of the experimental protocol. Identical 2 × 2 cm areas were demarcated on both the left (control) and right (PBM‐treated) arms. (1) Baseline period: Tape stripping was performed followed by initial fluorescence imaging and spectroscopy. (2) Preincubation: MAL cream was applied to both arms for 30 min. (3) PBM intervention: On the right arm only, PBM was applied immediately after the initial MAL application. (4) Main incubation: Excess cream was removed and MAL was reapplied to both arms for an additional 180 min. (5) Final assessment: Excess cream was removed and final fluorescence measurements were acquired from the same regions.

Before MAL application, tape stripping was performed on the marked skin area to remove excess dead cells from the stratum corneum, facilitating better cream penetration. Subsequently, a topical cream formulation containing 20% MAL—dimethyl sulfoxide (DMSO) content is below 10% (v/v) and the ethylenediaminetetraacetic acid (EDTA) content is below 5% (m/v)—(EMIPHARMA, EMBRAPII Project, São Carlos/SP, Brazil) was applied to the marked area (amount applied sufficient to cover the area of interest), which was then occluded with plastic film and aluminum foil for 30 min to maintain occlusion and prevent any light exposure during the incubation period. Approximately 1 g of MAL cream was used in total for each volunteer, distributed between both forearms and across the two applications Following the initial 30 min incubation with MAL, the excess cream was carefully removed with sterile gauze, and PBM was performed using either the Twin Flex Evolution (780 nm) or Premier Twin Flex (808 nm) device (MMOptics, São Carlos/SP, Brazil), both approved for clinical use. The laser beam spot size at the handpiece output was 4 mm^2^ for the 780 nm device and 3 mm^2^ for the 808 nm device. Fluences of 5, 10, or 15 J/cm^2^ were delivered depending on group assignment. In the right arm, PBM was applied by irradiating the selected area, after which MAL was reapplied to the same site and kept under occlusion for an additional 180 min [16], resulting in a total incubation time of 210 min. After this period, the excess cream was removed again, and PpIX production was assessed by fluorescence spectroscopy (spectrophotometer USB2000, OceanOptics Inc., USA) at 408 nm excitation and by fluorescence imaging. To ensure identical conditions, the same procedure of removing and reapplying MAL for 180 min was also performed on the left arm, which did not receive PBM (Figure 1). Fluorescence images were acquired using the LINCE imaging system (MMOptics, São Carlos/SP, Brazil) [17]. The LINCE device is a dual‐platform system designed for photodynamic applications. It integrates a wide‐field fluorescence imaging module, which employs a 405 nm LED array to excite fluorescence emissions above 450 nm, together with an illumination probe composed of 630 nm LEDs suitable for PDT treatment [17, 18]. In the present study, only the fluorescence imaging module was used. The acquisition time for both fluorescence spectra and images was a few seconds, which did not interfere with the experimental results.

**FIGURE 2:**
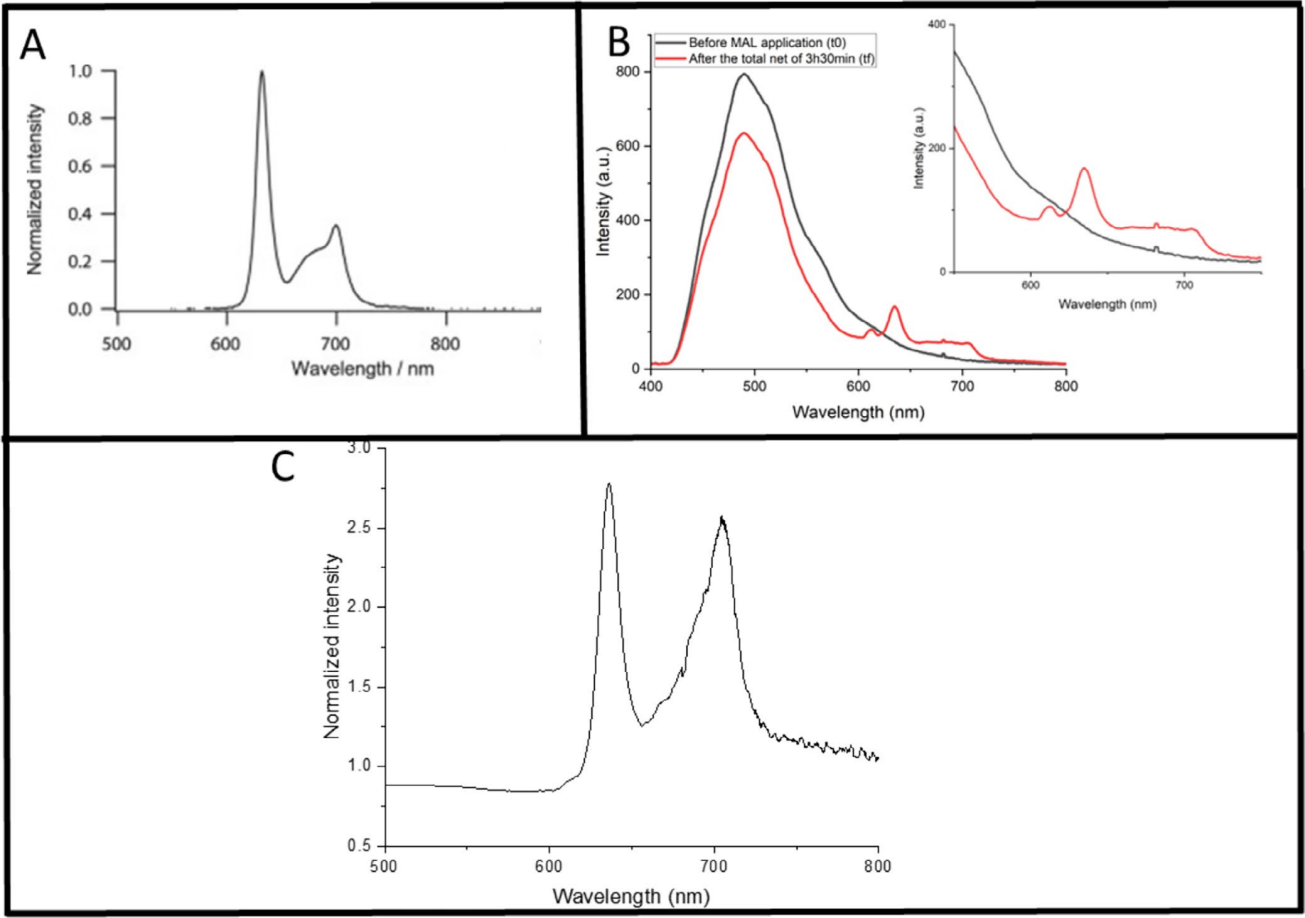
(A) Fluorescence spectra of protoporphyrin IX, highlighting the peaks at 635 nm and 700 nm (adapted from Takeo Minamikawa et. al) [19]. (B) Average fluorescence spectra obtained before MAL application (baseline, black line) and after 210 min of incubation with MAL (final timepoint, red line). The spectra shown correspond to one representative volunteer. A zoomed view of the 550–750 nm region is provided to better visualize the characteristic PpIX peaks. (C) Spectrum obtained by dividing the average spectrum at the final timepoint, (tf) by the average baseline spectrum, (t0) to quantify the increase in fluorescence intensity at 635 and 700 nm.

At baseline (t0), defined as the initial time point before MAL application, four fluorescence spectra were collected from the corners of the marked square on each forearm (control and PBM‐treated). The spectra from each arm were averaged to obtain a representative baseline value per volunteer. After the total incubation period of 210 min, spectra were collected again from the same four corner points, plus one additional spectrum from the center of the square, and averaged in the same way (Figure 2B). To quantify the increase in fluorescence signal with time, the mean post‐incubation spectrum was divided by the mean baseline spectrum (t0) (Figure 2C). The analysis focused on the characteristic PpIX fluorescence peaks at approximately 635 and 700 nm. For each individual, this calculation provided values for both arms (left arm without PBM and right arm with PBM). To determine whether PBM enhanced PpIX production, an additional ratio was calculated by dividing the PBM‐treated arm values by those of the control arm (PBM/no PBM).

The images were processed using a Python‐based algorithm that calculates the average pixel intensity of the red component in the RGB system, which was interpreted as a measure of PpIX fluorescence. Specifically, the *roi_stats* function from the Image Functions Library, available at github.com/MarlonGarcia/imfun, was used. This library is designed for image manipulation and preprocessing. Spectroscopic and image‐based analyses were performed independently and subsequently compared to assess consistency between the two methods.

An additional analysis was conducted to evaluate the relationship between PpIX production and the volunteers' skin type, classified according to the Fitzpatrick scale [20], which categorizes human skin phototypes based on pigmentation, sensitivity to ultraviolet (UV) radiation, and tanning response. Only volunteers with skin types I–IV were included in this study: type I (very fair skin, always burns and never tans), type II (fair skin, usually burns and tans minimally), type III (light to medium skin, sometimes burns but tans uniformly), and type IV (olive or moderate brown skin, rarely burns and tans easily). Furthermore, we also examined the influence of ancestry by correlating self‐identified skin color and PpIX production.

In addition to the experiments on healthy volunteers, a representative in vivo case of PDT for actinic keratosis was included to illustrate the translational potential of the proposed protocol. The procedure was performed as follows: (1) mechanical shaving of the treatment area with a sterile scalpel; (2) application of 20% ALA cream, followed by a 30‐min incubation period under occlusion; (3) irradiation with near‐infrared light (*λ* = 850 nm, *t* = 10 min, Fluence = 60 J/cm^2^) over the entire lesion area; (4) reapplication of 20% ALA and continued incubation for a total of 180 min; and (5) PDT (*λ* = 630 nm, *t* = 20 min, Fluence = 150 J/cm^2^) initiated 180 min after ALA reapplication. For comparison, a contralateral lesion from the same patient was treated with the identical PDT protocol but without prior PBM. To obtain the images and apply PDT, the same LINCE device described was used.

Data were analyzed using nonparametric statistics due to the small sample size (*n* = 5 per group). The Wilcoxon signed‐rank test was employed to determine if the PBM/no‐PBM ratios differed significantly from unity (1.0). For comparisons between multiple fluences and wavelengths, the Kruskal‐Wallis test followed by Dunn's post hoc test was used. Results are expressed as mean ± standard deviation (SD), and interindividual variability was further quantified using the coefficient of variation (CV). Differences were considered statistically significant at *p* ≤ 0.05. All statistical analyses were performed using GraphPad Prism (GraphPad Software, San Diego, CA, USA).

## 3 Results

**FIGURE 3:**
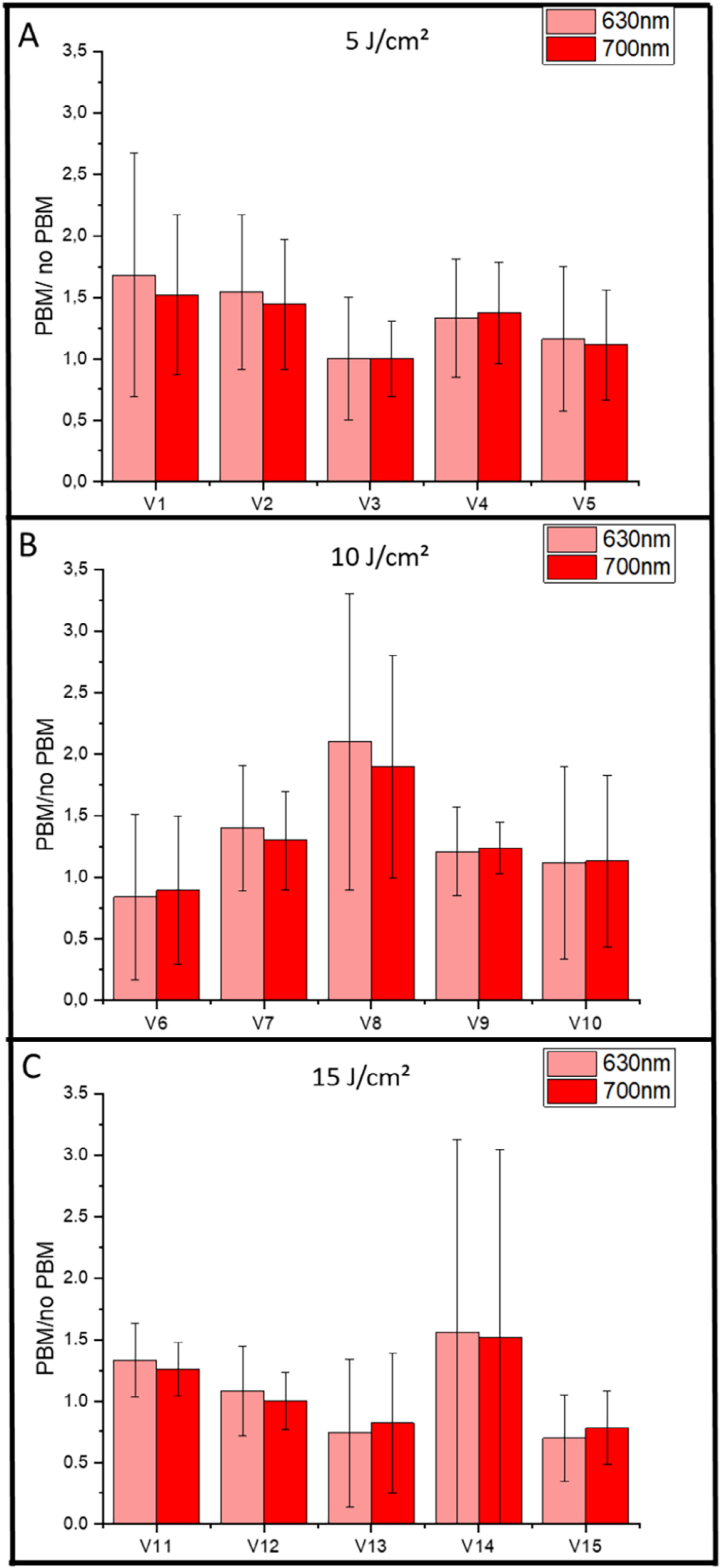
Results from all volunteers, summarized across panels A, B, and C. Individual group results are shown for: G1 (780 nm, 5 J/cm^2^), G2 (780 nm, 10 J/cm^2^), and G3 (780 nm, 15 J/cm^2^). Data represent the PBM/no PBM ratio for groups treated with PBM at 780 nm. Values correspond to the mean spectroscopy measurements at the characteristic PpIX fluorescence peaks (635 and 700 nm), and error bars indicate standard deviations.

Fluorescence spectroscopy and image‐based quantification using the LINCE system were employed to evaluate potential variations in PpIX production in response to PBM at 780 nm. Spectra were obtained for the different light fluences (5, 10, and 15 J/cm^2^), and the PpIX peaks at 635 and 700 nm were analyzed. For each group of five volunteers, the signal intensity was measured before and after topical MAL application, comparing the arm treated with PBM and the untreated control arm. The fluorescence ratio (PBM/no PBM) was calculated for both wavelengths. In this exploratory analysis, ratios above 1 were considered indicative of a positive trend toward enhanced PpIX production, while a formal inferential test against unity was performed to assess significance (Figure 3).

Across all groups, mean PBM/no PBM ratios were nominally greater than 1. While these means might suggest a trend toward increased PpIX accumulation in the PBM‐treated region, no statistically significant differences were observed when comparing these ratios to the null hypothesis (ratio = 1) or between groups (*p* > 0.05). This lack of significance is likely due to the considerable interindividual variability observed in the spectroscopic data, where the highest average ratio was noted at 10 J/cm^2^ but without statistical support for superiority.

**FIGURE 4:**
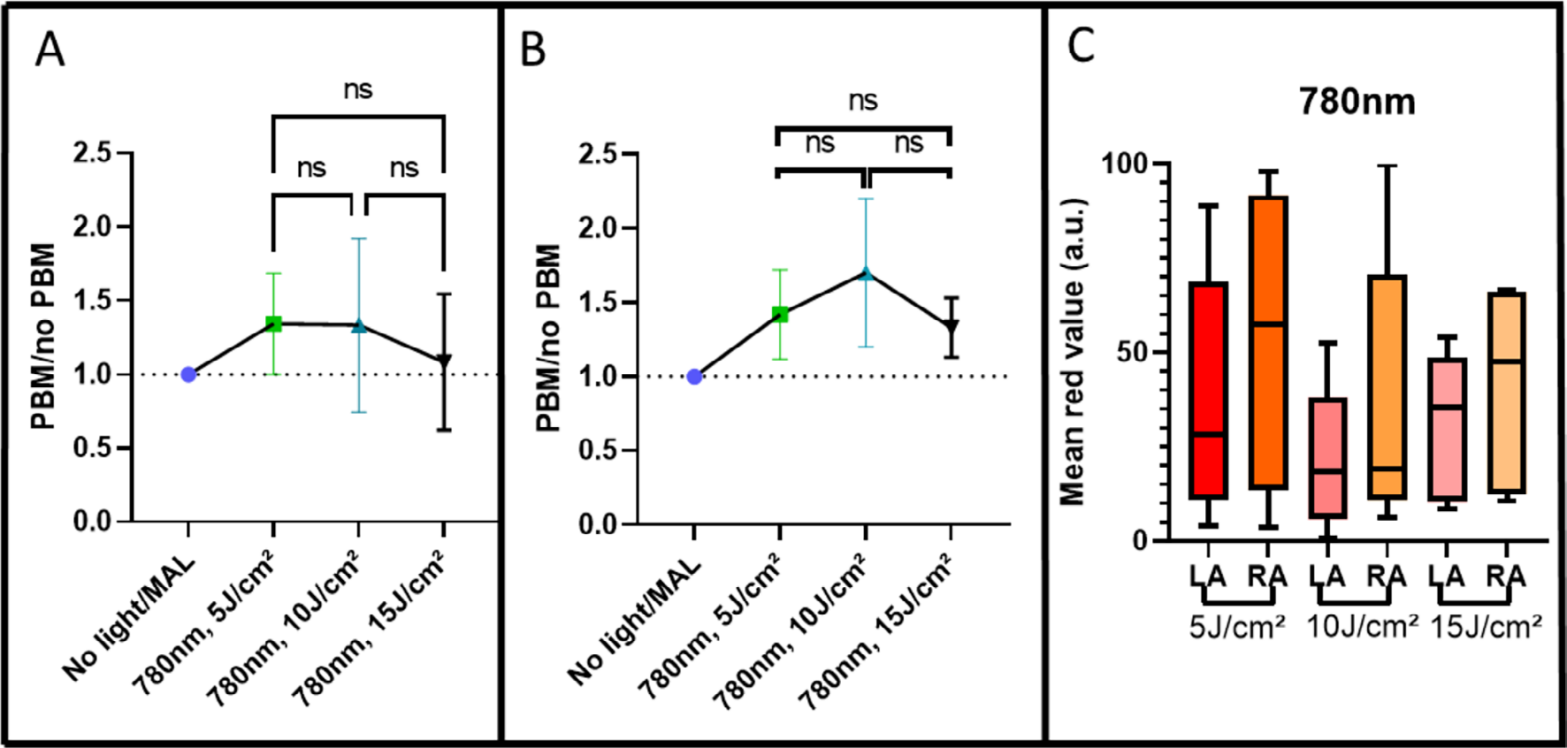
(A) Mean PBM/no PBM ratio values at 635 nm for PBM at 780 nm for spectroscopic measurements; no statistically significant differences were observed among groups. (B) Mean PBM/no PBM ratio values for fluorescence images acquired with the LINCE system; no statistically significant differences were observed among groups. (C) Boxplot showing the fluorescence signal intensity from fluorescence images before and after topical MAL application combined with PBM using fluences of 5, 10, and 15 J/cm^2^, where LA is left arm and RA is right arm. Statistical analysis was performed using Kruskal‐Wallis test (*p* ≤ 0.05) and error bars indicate 95% CI. Interindividual variability was assessed via the coefficient of variation (CV).

The graphs in Figure 3 were constructed by normalizing the fluorescence in the PBM‐treated region to that of the untreated region, creating a dimensionless scale. A value of 1 indicates no change. While values above 1 suggest an increase in PpIX, they must be interpreted as exploratory trends given the high dispersion of the data. Although the majority of volunteers showed a positive nominal response, with an average observed increase of approximately 30%–40% (Figure 4A), the high coefficient of variation highlights the heterogeneity of the biological response.

In contrast, fluorescence image analysis (Figure 4B) suggested a possible dose dependent trend: the group irradiated with 10 J/cm^2^ presented the highest mean ratio (~1.7). However, despite the visually higher mean and a lower standard error in this group, the differences between all three fluences (5, 10, and 15 J/cm^2^) were not statistically significant. These findings highlight the complementary nature of both evaluation methods but emphasize that 10 J/cm^2^ at 780 nm should be viewed only as a potential candidate fluence for further investigation in a larger cohort.

In general, for 780 nm, spectroscopic measurements revealed wide interindividual variability at all fluences, as shown by the standard deviation across subjects (Figure 4A). The interindividual variability was substantial across all groups, with coefficients of variation (CV) of 20.53% for 5 J/cm^2^, 35.49% for 10 J/cm^2^, and 34.36% for 15 J/cm^2^. Due to this dispersion, a one‐sample Wilcoxon test against unity (H_0_ = 1.0) revealed no statistically significant differences (*p* > 0.05), suggesting that the observed increases represent exploratory trends rather than a uniform clinical effect.

In contrast, image‐based analysis using the LINCE system indicated that the 15 J/cm^2^ fluence appeared to result in a more visually homogeneous PpIX distribution across individuals (Figure 4B). While 5 J/cm^2^ produced high mean values in some cases, its variability was notably greater (Figure 4C). These observations suggest that higher fluences might contribute to a more uniform photosensitizer accumulation, although this remains to be confirmed by higher‐powered studies.

**FIGURE 5:**
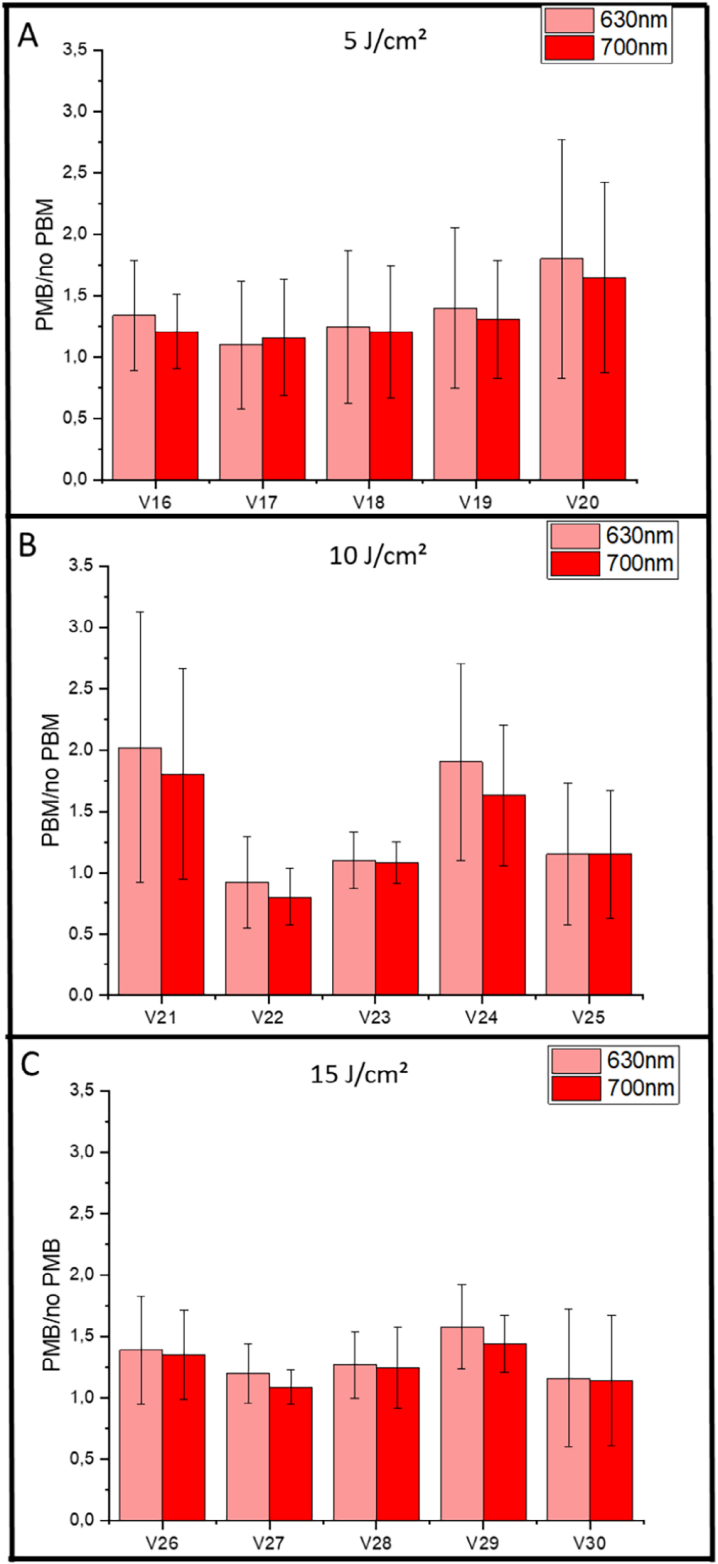
Results from all volunteers, divided into panels A, B, and C. Individual group results are shown for: G1 (808 nm, 5 J/cm^2^), G2 (808 nm, 10 J/cm^2^), and G3 (808 nm, 15 J/cm^2^). Data represent the PBM/no PBM ratio for groups treated with PBM at 808 nm. Values correspond to the mean spectroscopy measurements at the characteristic PpIX fluorescence peaks (635 and 700 nm), and error bars indicate standard deviations.

**FIGURE 6:**
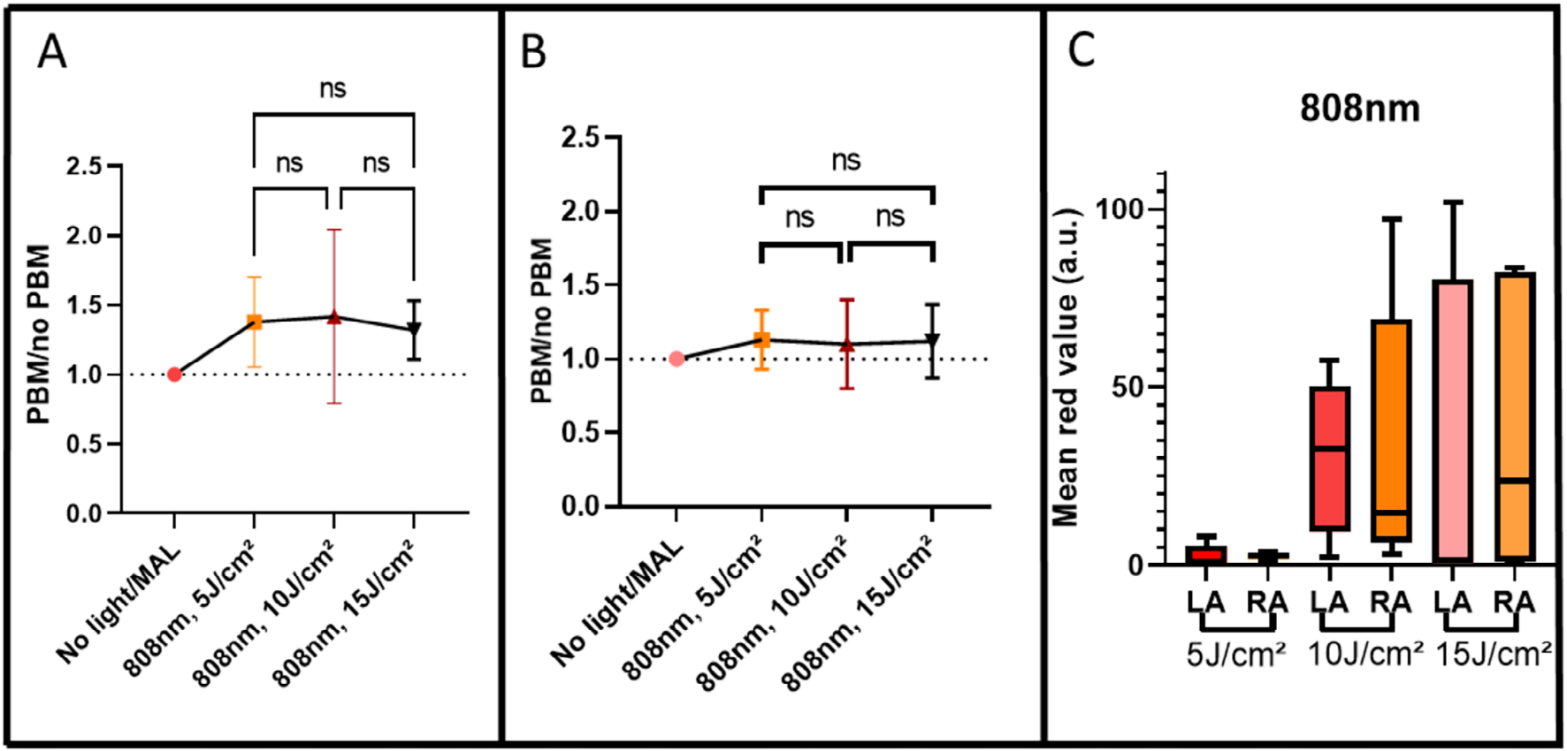
(A) Mean PBM/no PBM ratio values at 635 nm for PBM at 808 nm for spectroscopic measurements; no statistically significant differences were observed among groups. (B) Mean PBM/no PBM ratio values for fluorescence images acquired with the LINCE system; no statistically significant differences were observed among groups. (C) Boxplot showing the fluorescence signal intensity from fluorescence images before and after topical MAL application combined with PBM using fluences of 5, 10, and 15 J/cm^2^. Statistical analysis was performed using the Kruskal‐Wallis test (*p* ≤ 0.05), and error bars indicate 95% CI. Interindividual variability was assessed via the coefficient of variation (CV).

Spectroscopic analysis of PpIX fluorescence at 808 nm revealed that at 5 J/cm^2^, most participants exhibited only nominal increases in PBM/no PBM ratio, with a coefficient of variation of 18.98%. In contrast, 10 J/cm^2^ resulted in higher average observed PpIX production in several subjects, although interindividual variability was most pronounced at this fluence (CV = 35.54%). At 15 J/cm^2^, the magnitude of the response appeared more moderate, but the data distribution was narrower, as evidenced by the lowest CV among all groups (12.85%), suggesting a trend toward more consistent outcomes across the group (Figure 5). Among the tested fluences for 808 nm, 10 J/cm^2^ showed the highest mean ratio; however, these differences were not statistically significant (*p* > 0.05), and an inferential test against unity did not confirm a conclusive enhancement effect (Figure 6A).

In contrast, image‐based analysis using the LINCE system demonstrated more subtle changes in PpIX fluorescence intensity across all fluences, with values near to 1.0 and no clear fluence‐response trend (Figure 6B). When comparing spectroscopy and imaging data, spectroscopy appeared to show a numerically stronger signal, with mean PBM/no PBM ratios of 1.3 and 1.4, whereas image analysis yielded values closer to 1.1 across all tested fluences. Although no statistically significant differences were found between groups or analytical methods, a subtle exploratory trend toward increased PpIX signal was observed. The discrepancy in variability—where 10 J/cm^2^ showed the highest dispersion and 15 J/cm^2^ the greatest homogeneity—highlights the complex nature of light‐tissue interaction at this wavelength.

Image‐based analysis suggested that the fluorescence signal at 5 J/cm^2^ remained largely at baseline levels, whereas 10 J/cm^2^ led to a nominal signal enhancement in some individuals, albeit with considerable variability. At 15 J/cm^2^, the observed fluorescence increase was moderate but showed a tighter distribution, potentially indicating greater homogeneity. Together, these findings suggest that while 10 J/cm^2^ might induce stronger PpIX synthesis in select individuals, the 15 J/cm^2^ fluence appears to promote a more uniform and reproducible response across participants, which may be clinically relevant for standardized protocols.

**FIGURE 7:**
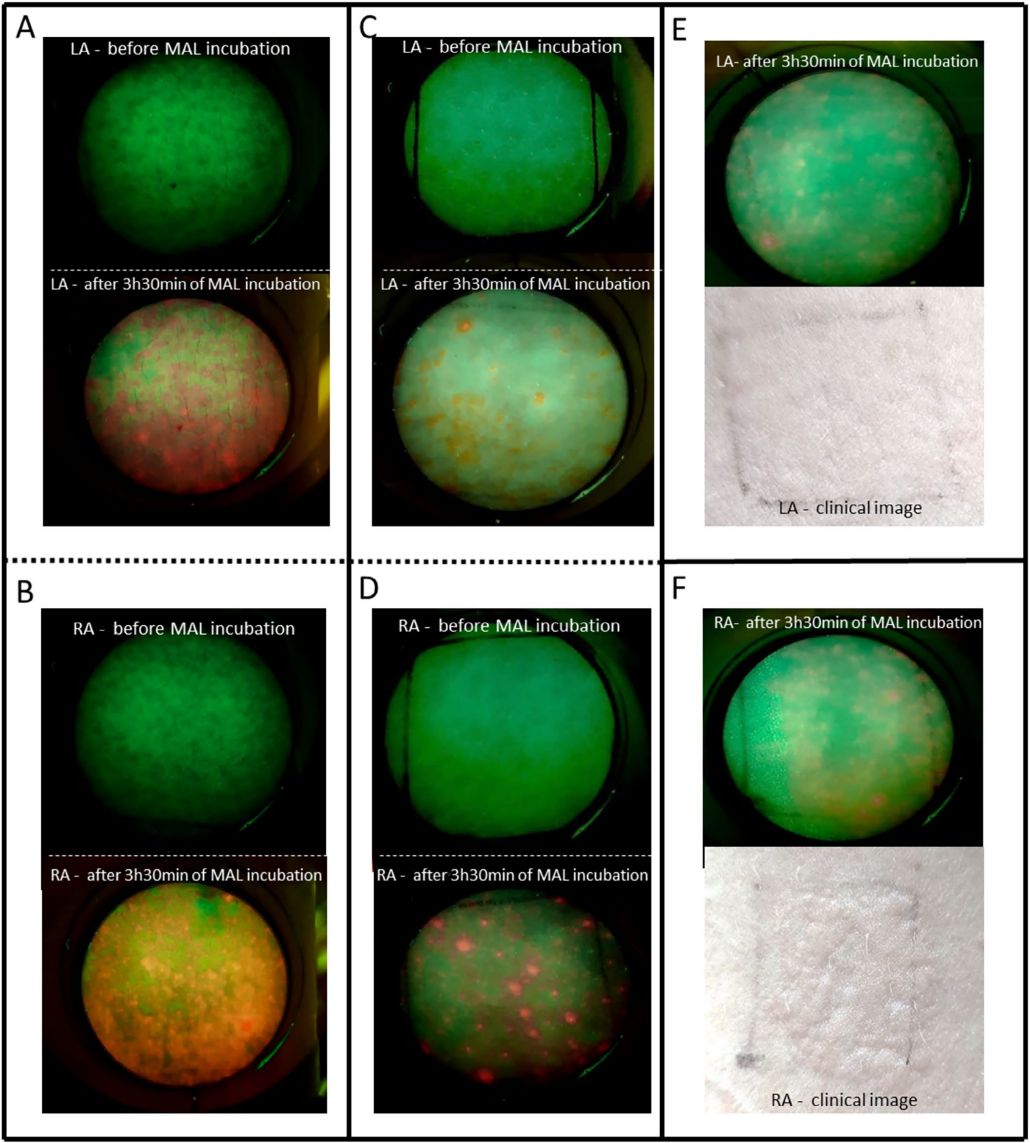
Representative fluorescence and clinical images of normal skin from healthy volunteers. (A and C) Fluorescence images of the left arm (LA, control) at baseline and after 210 min of MAL incubation. (B and D) Fluorescence images of the right arm (RA, PBM‐treated, 808 nm, 15 J/cm^2^) at baseline and after 210 min of MAL incubation. A/B and C/D represent two different volunteers, showing interindividual variability. (E) Post‐incubation fluorescence and clinical photograph of the control arm. (F) Post‐incubation fluorescence and clinical photograph of the PBM‐treated arm, exhibiting a more visible erythemal response (skin reaction) as a qualitative indicator of enhanced activity.

As shown in Figure 7, the response at 808 nm with 15 J/cm^2^ appears visually more homogeneous. In the left arm (Figure 7A,C), fluorescence was observed to be more widely distributed compared to the control. Additionally, clinical observations of the skin reaction in the right arm (Figure 7B,D) may provide qualitative support for increased PpIX activity, although these findings remain descriptive and lack statistical confirmation of superiority over the control arm (Figure 7E,F).

**FIGURE 8:**
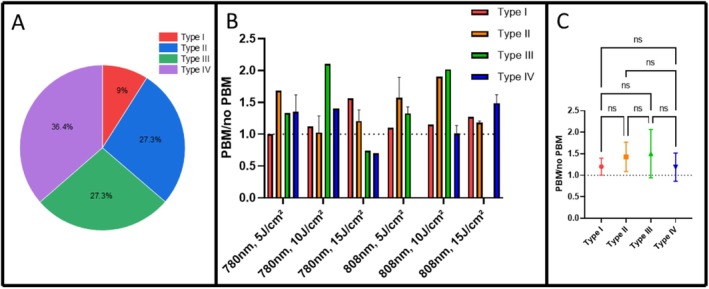
(A) Percentage distribution of volunteers according to the Fitzpatrick skin type classification (types I–IV). (B) Mean PBM/no PBM ratios for each skin type across all groups and fluences, obtained from fluorescence spectroscopy data. (C) Average PBM/no PBM ratios by Fitzpatrick skin type (mean ± SD). No statistically significant differences were found between groups (ns, *p* > 0.05).

An analysis based on the Fitzpatrick skin type classification was performed, with the volunteers ranging from types I to IV (Figure 8). Although increased PpIX production was observed across all groups, the magnitude of the mean increase was numerically greater in skin types II and III (Figure 8B,C).

**FIGURE 9:**
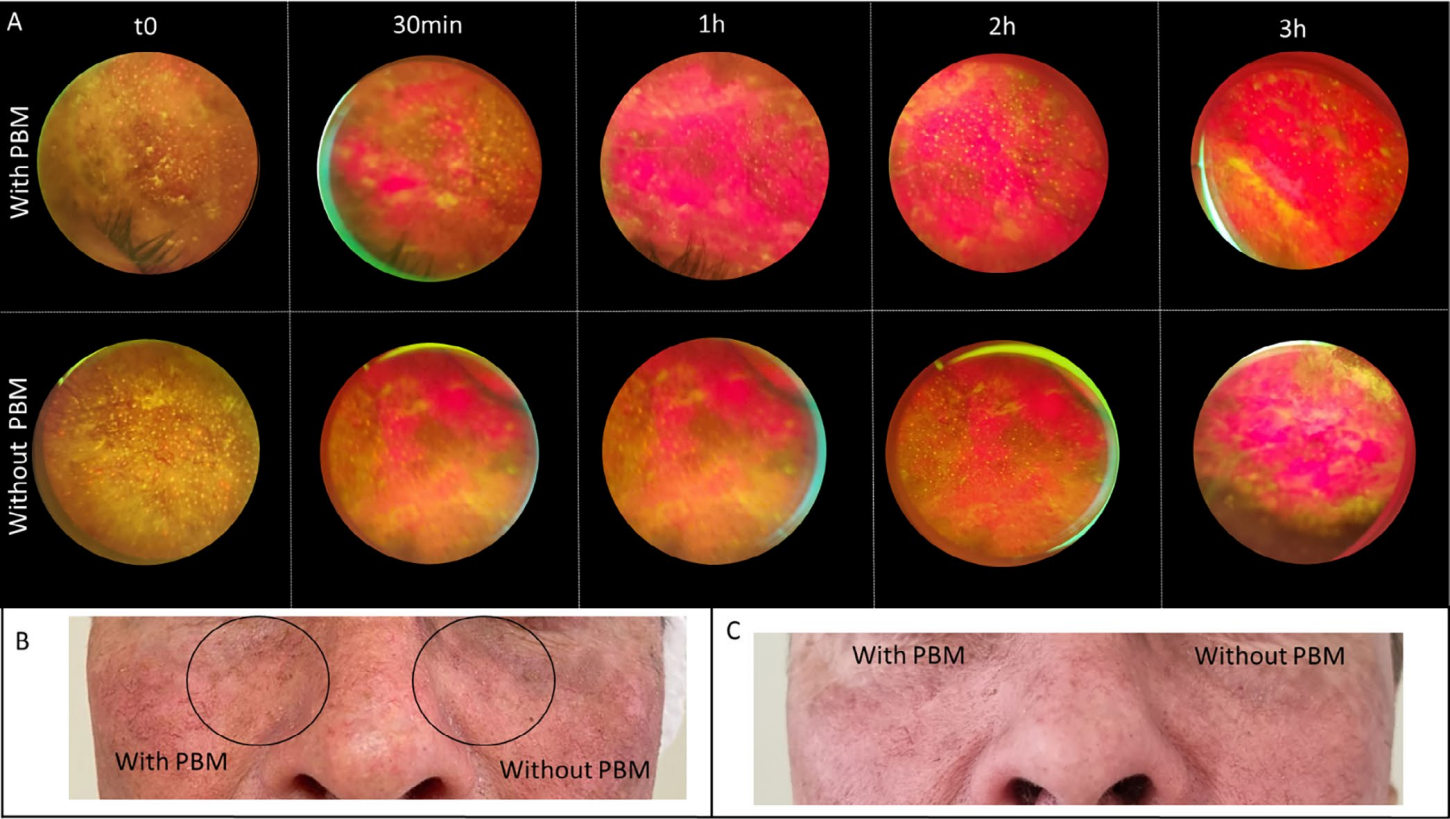
Clinical application of PBM‐enhanced PDT in a patient with Actinic Keratosis (AK) lesions. (A) Real‐time fluorescence imaging of two distinct AK lesions: One pretreated with PBM (850 nm, 60 J/cm^2^) and the other no PBM, showing the progression of PpIX accumulation. (B) Baseline clinical appearance of the AK lesions. (C) Clinical follow‐up 30 days post‐treatment, demonstrating the therapeutic outcome (lesion clearance) in both areas.

To explore the clinical relevance of these findings, a case study was conducted on a patient with Actinic Keratosis (AK) (Figure 9). Although the previous experiments in normal skin showed high interindividual variability and no statistical significance, the clinical application in AK lesions suggested a potential benefit of PBM preirradiation. As shown in Figure 9A, the lesion exhibited a visually more intense PpIX fluorescence signal compared to the lesion without PBM. While both lesions showed clinical improvement after 30 days (Figure 9C), this preliminary observation aligns with the exploratory trends noted in the healthy volunteer groups, where specific fluences (10–15 J/cm^2^) appeared to facilitate a more pronounced photosensitizer accumulation. However, it is important to note that these clinical findings are descriptive and require further validation in controlled clinical trials with larger sample sizes.

## 4 Discussion

This study investigated the effects of PBM using NIR light at 780 and 808 nm on the accumulation of PpIX after topical application of MAL. The rationale behind combining PBM with MAL application lies in the ability of PBM to modulate mitochondrial activity, potentially enhancing the synthesis of PpIX, which occurs endogenously in the mitochondria as part of the heme biosynthetic pathway [4, 5].

Both 780 and 808 nm wavelengths lie within the near‐infrared (NIR) region, where light penetration into skin is relatively high. According to Finlayson et al. [21], the effective penetration depths of 780 and 808 nm light in skin are very similar, which may explain why no major differences were observed between the two wavelengths in our results. Therefore, our comparison of 780 and 808 nm PBM primarily aimed to assess potential variations in mitochondrial stimulation and PpIX biosynthesis under two commonly used NIR conditions, rather than to probe depth‐dependent effects.

Although no statistically significant differences were found between the tested fluences, the mean PBM/no PBM ratio was consistently above 1 across all groups. While this suggests a nominal increase in PpIX production, these findings should be interpreted as exploratory trends rather than conclusive evidence of enhancement, given the lack of inferential significance against unity. Nevertheless, these preliminary observations in normal skin provide a rationale for further investigating whether PBM could potentiate PpIX production in clinical PDT protocols.

Spectroscopic fluorescence analysis showed that the PBM/MAL combination led to an increase in PpIX signal compared to the nonirradiated control (PBM/no PBM ratio > 1) in most volunteers. Complementary qualitative analysis using fluorescence images acquired with the LINCE system further supports this observation.

It is important to note that in some volunteers (approximately 27%), the PBM/no PBM ratio for 780 nm was below 1.0. This notable interindividual variation (with coefficients of variation occasionally exceeding 40%) is expected in human studies. Factors such as hydration level, vascularization, oxygenation, sebaceous activity, and even local asymmetries between the two arms can influence MAL penetration and PpIX synthesis. Furthermore, the contribution of skin phototype cannot be disregarded; our data suggests that melanin content and ancestry (e.g., participants of Japanese vs. European descent) may influence light‐tissue interaction, potentially requiring personalized PBM dosing rather than a standardized approach to achieve optimal mitochondrial stimulation.

The comparative analysis revealed that both 780 and 808 nm wavelengths yielded mean ratios above 1. At 808 nm, while 10 J/cm^2^ showed the highest nominal mean in spectroscopy, it was also associated with the greatest interindividual variability (CV = 35.54%), reducing its predictability. In contrast, 15 J/cm^2^ appeared to produce a more modest but consistent response, particularly in fluorescence imaging (CV = 12.85%). This suggests that while lower fluences might induce stronger synthesis in “high‐responders,” higher fluences could promote more uniform photosensitizer distribution, a key factor for the clinical efficacy and reliability of PDT.

The apparent reduction in detected PpIX signal at 15 J/cm^2^ in some cases may be explained by nonexclusive mechanisms. First, PBM classically exhibits a biphasic dose–response (hormetic) in which supra‐optimal fluences can reduce stimulation [22, 23]. Second, the possibility of PpIX photobleaching during or immediately after high‐energy irradiation could reduce measurable fluorescence [24]. Finally, measurement or sampling effects (e.g., interindividual heterogeneity, detector saturation, or slight differences in probe positioning) may contribute to the observed decrease. Future work should include temperature monitoring, time‐course sampling immediately post‐PBM to assess photobleaching kinetics, and larger cohorts to reduce the impact of individual variability.

Andrea Lesar et al. investigated PpIX fluorescence after the application of ALA‐ and MAL‐containing creams on normal skin [16]. They obtained fluorescence signal curves over time and demonstrated that a clear fluorescence signal could already be observed after 180 min of MAL application [16]. This indicates that such an incubation period is clinically feasible, particularly for PDT. Since fluorescence is already evident in normal skin, an even stronger signal can be expected in precancerous or malignant lesions.

Our findings align with previous preclinical results, including the study by Joniová et al. which demonstrated that PBM could stimulate and homogenize PpIX production in glioma cells in vitro and tumors grown on the chicken embryo chorioallantoic membrane (CAM) model. In their protocol, PBM was applied at 652, 730, or 808 nm with an irradiance of 3 mW/cm^2^ for 180 s, followed by administration of ALA 24 h later and fluorescence imaging after a 4‐h incubation period [11]. While their work focused on malignant cells and a delayed ALA protocol, our study was designed as an initial proof‐of‐concept to determine whether PBM could enhance PpIX production in normal human skin. Our protocol, which involved MAL application followed by PBM and subsequent reapplication of MAL, demonstrated a modest but consistent increase in PpIX fluorescence. However, we do not claim that this sequence is necessarily optimal. Alternative approaches, such as applying PBM before or after different incubation times with MAL, or even testing PBM without reapplication, could potentially yield stronger effects. Future studies should systematically compare these different strategies to identify the most effective protocol for maximizing PpIX production. Nonetheless, the present results highlight the promise of PBM as an adjuvant for PDT and provide a rationale for further protocol optimization.

Even though the exact mechanisms of PBM within the mitochondria are still being researched, it is known to boost mitochondrial activity, leading to an overall increase in a cell's metabolism, mainly by increasing ATP synthesis [1, 2]. Studies have also shown that PBM increases mitochondrial permeability [25], leading to greater production of ROS [26, 27], and the release of nitric oxide (NO) [28]. NO is a key molecule involved in crucial cellular functions like cell proliferation, differentiation [29], and inflammatory responses [29, 30]. Additionally, NO has been found to increase the photosensitivity of cells treated with ALA [31]. This effect is associated with a decrease in the activity of the FECH enzyme, which in turn causes the accumulation of PpIX [31]. Based on these findings, we can hypothesize that PBM might enhance the biosynthesis and distribution of PpIX in tissue through two potential pathways: (a) by enhancing mitochondrial activity and permeability: This could accelerate PpIX production, but could also result in heme production, thus acting against PpIX accumulation; (b) by increasing NO levels: This is a more direct pathway, as NO has been linked to decreased FECH activity, which stimulates the accumulation of PpIX [31].

Although no statistically significant differences were found between groups due to sample size and variability, the observed trends suggest a promising role for PBM in optimizing PpIX‐based therapies. These results may also have implications in the field of dermatology, where enhancing mitochondrial activity and uniform photosensitizer distribution could support noninvasive skin rejuvenation and other light‐based treatment strategies.

One of the main questions that arises from this study is how this protocol would behave in tumors, as we only applied it to healthy skin. Studies have shown that cancer cells naturally accumulate more PpIX than healthy cells, which is due to a decrease in FECH activity and an increase in ALA uptake [32]. While PpIX accumulation isn't significant in healthy cells, the inclusion of DMSO and EDTA in our topical formulation enhances PpIX accumulation even in normal skin [33]. The benefits of using PBM to produce PpIX are clear, but a valid concern is that PBM can also stimulate cell proliferation, which could be worrisome in the case of tumors [34]. However, this concern is mitigated by the fact that the PDT protocol, which is applied immediately after PBM, should induce cell destruction that far outweighs any potential increase in cell multiplication, which is typically a slower process. Future studies using animal models are needed to explore the mechanistic aspects of this protocol and to determine if similar enhancements are observed in diseased skin or neoplastic lesions. This research could potentially pave the way for refined PDT protocols that integrate PBM to improve both efficacy and selectivity.

An additional analysis was conducted to evaluate whether the Fitzpatrick skin type influenced PpIX production following PBM. Figure 8 shows the distribution of the volunteers (types I–IV) and the corresponding mean PBM/no PBM ratios obtained from fluorescence spectroscopy data. Although no statistically significant differences were detected among skin types, the mean ratios were consistently above 1 for all groups, indicating that PBM tended to enhance PpIX production regardless of skin pigmentation. Interestingly, volunteers with type II and III skin exhibited slightly higher average ratios, suggesting a possible balance between light penetration and melanin absorption that could favor PBM‐induced mitochondrial stimulation.

In type IV skin, the greater melanin content may have absorbed a significant portion of the PBM light, reducing its effect. In contrast, in type I skin, the lack of pigmentation may not have substantially influenced cellular metabolism, or the skin may have reached a saturation point in PpIX production more rapidly. It is also noteworthy to consider the ancestry of the volunteers, most of whom were of European origin (Portuguese, Spanish, Italian, French), but also included Japanese, Black, Indigenous from Brazil and Peru (Inca), as well as mixed Brazilian backgrounds. We observed a trend in which participants of Japanese descent exhibited higher PpIX production compared to the others, while those with lighter skin, predominantly of European ancestry, tended to produce less.

These findings represent only a preliminary indication, as the present study was not specifically designed to assess this variable in depth. However, the observed tendency highlights an important direction for future research aimed at understanding how skin phototype may influence PBM‐mediated PpIX synthesis.

Another factor not addressed in this work is the issue of pain following topical PDT protocols. During PDT, patients often report pain in the initial minutes due to the oxidative effect on nerve endings. In clinical pilot studies that are already underway, because PBM reduces inflammation [35], the pain appears to be significantly reduced, demonstrating an additional important benefit of incorporating PBM into dermatological PDT [36, 37]. Moreover, the results demonstrate that it is not only about increasing PpIX production but ensuring its uniform distribution across the skin, a key factor for the clinical efficacy of PDT. The homogeneity observed, particularly at 15 J/cm^2^, suggests that it is possible to combine treatment intensity with consistency, paving the way for personalized and safer protocols.

It is important to recognize that the fluorescence spectroscopy and imaging techniques used in this study, which rely on excitation in the 400–450 nm range, are limited to detecting PpIX within approximately 0.5 mm of the skin surface [21, 38, 39]. Consequently, the detected fluorescence mainly reflects accumulation in the epidermis and upper dermis. It is also conceivable that near‐infrared irradiation (780 and 808 nm) may also stimulate mitochondrial activity and PpIX production in deeper layers, beyond the detection depth of our system. To explore this hypothesis, our group has initiated studies using tumor‐bearing animal models to extract and quantify PpIX from different tissue depths following PBM. These experiments will help determine whether PBM enhances PpIX production in deeper tumor layers, potentially improving the therapeutic efficiency of PDT.

Although our dataset included only two time points (baseline and 210 min), it is possible to make a preliminary estimation of the potential acceleration in PpIX accumulation induced by PBM. Using the mean normalized fluorescence values (control arm, LA = 2.85 ± 2.3; PBM‐treated arm, RA = 3.18 ± 1.9), and assuming a linear increase in signal over time, the control site would be expected to reach the same fluorescence level as the PBM‐treated site after approximately 4.1 h of incubation. This corresponds to a nominal reduction of about 37 min in the effective incubation time attributable to PBM.

It is important to note that this calculation is purely indicative, based on simplified assumptions and limited data, and should not be interpreted as a definitive kinetic model. Nonetheless, it provides an initial quantitative perspective on how PBM may accelerate PpIX production, warranting future studies with additional sampling points and refined kinetic analysis.

It is also important to acknowledge that the present study was conducted on normal skin, which differs from neoplastic or precancerous tissue in metabolic rate, vascular supply, oxygenation, and permeability. PDT involves the interplay of three fundamental components—light, photosensitizer, and oxygen—and the present findings specifically address the photosensitizer aspect, showing that PBM can modulate PpIX synthesis through mitochondrial stimulation. In neoplastic tissue, where mitochondrial metabolism and oxygen consumption are altered, these effects may be further amplified or modified.

Although this study focused on normal skin, the observed PBM‐induced increase in PpIX production may also have implications for esthetic dermatology. PDT has already been used in skin rejuvenation protocols, where its photobiological effects promote collagen remodeling and tissue renewal [40, 41, 42, 43]. By stimulating mitochondrial metabolism and enhancing PpIX synthesis, PBM could potentially reduce the required photosensitizer incubation time while maintaining or even improving treatment efficacy.

The addition of a clinical case involving actinic keratosis illustrates the exploratory potential of PBM‐assisted PDT. Although the sample size (*n* = 5 per group) and biological variability limit the interpretability of the healthy volunteer data, the qualitative increase in PpIX fluorescence and the observed clinical outcome in this case study are encouraging. These preliminary findings lay the groundwork for future clinical trials to determine if the observed trends translate into improved therapeutic efficacy in neoplastic lesions.

## 5 Conclusion

This study provides preliminary indications that PBM with near‐infrared light at 780 and 808 nm may influence PpIX accumulation in normal human skin following topical application of MAL. Although no statistically significant differences were found among fluences or wavelengths, the mean PBM/no PBM ratios were above 1 across all groups, suggesting an exploratory trend toward increased PpIX fluorescence. Both spectroscopy and fluorescence imaging consistently showed this pattern; while lower energy fluences tended to yield slightly higher mean values, higher fluences (specifically 15 J/cm^2^) appeared to promote greater spatial uniformity and lower interindividual variability.

These findings point to PBM as a potential adjuvant strategy to optimize PDT, offering a noninvasive pathway to modulate PpIX production. To the best of our knowledge, this is the first report exploring the combination of PBM and MAL in normal human skin. Given the high biological variability observed, future studies with larger cohorts are necessary to validate these benefits in pathological skin and therapeutic contexts, paving the way for more personalized and predictable PDT protocols.

## Author Contributions

**Letícia Palombo Martinelli:** conceptualization, data curation, formal analysis, investigation, methodology, project administration, validation, visualization, writing – original draft, writing – review and editing. **Natasha Ferreira Mezzacappo:** formal analysis, investigation, writing – review and editing. **Ana Paula Silva:** formal analysis, validation, writing – original draft, writing – review and editing. **Priscila Menezes:** investigation, methodology, writing – review and editing. **Vanderlei Salvador Bagnato:** conceptualization, formal analysis, funding acquisition, investigation, methodology, resources, supervision, validation, writing – original draft, writing – review and editing.

## Conflicts of Interest

The authors declare no conflicts of interest.

## Data Availability

The data that support the findings of this study are available from the corresponding author upon reasonable request.
